# Structure and Genome Release Mechanism of the Human Cardiovirus Saffold Virus 3

**DOI:** 10.1128/JVI.00746-16

**Published:** 2016-08-12

**Authors:** Edukondalu Mullapudi, Jiří Nováček, Lenka Pálková, Pavel Kulich, A. Michael Lindberg, Frank J. M. van Kuppeveld, Pavel Plevka

**Affiliations:** a Central European Institute of Technology, Masaryk University, Brno, Czech Republic; b Veterinary Research Institute, Brno, Czech Republic; c Department of Chemistry and Biomedical Sciences, Linnaeus University, Kalmar, Sweden; d Department of Medical Microbiology, Virology Section, Radboud University Nijmegen Medical Centre, Nijmegen, The Netherlands; Instituto de Biotecnologia/UNAM

## Abstract

In order to initiate an infection, viruses need to deliver their genomes into cells. This involves uncoating the genome and transporting it to the cytoplasm. The process of genome delivery is not well understood for nonenveloped viruses. We address this gap in our current knowledge by studying the uncoating of the nonenveloped human cardiovirus Saffold virus 3 (SAFV-3) of the family Picornaviridae. SAFVs cause diseases ranging from gastrointestinal disorders to meningitis. We present a structure of a native SAFV-3 virion determined to 2.5 Å by X-ray crystallography and an 11-Å-resolution cryo-electron microscopy reconstruction of an “altered” particle that is primed for genome release. The altered particles are expanded relative to the native virus and contain pores in the capsid that might serve as channels for the release of VP4 subunits, N termini of VP1, and the RNA genome. Unlike in the related enteroviruses, pores in SAFV-3 are located roughly between the icosahedral 3- and 5-fold axes at an interface formed by two VP1 and one VP3 subunit. Furthermore, in native conditions many cardioviruses contain a disulfide bond formed by cysteines that are separated by just one residue. The disulfide bond is located in a surface loop of VP3. We determined the structure of the SAFV-3 virion in which the disulfide bonds are reduced. Disruption of the bond had minimal effect on the structure of the loop, but it increased the stability and decreased the infectivity of the virus. Therefore, compounds specifically disrupting or binding to the disulfide bond might limit SAFV infection.

**IMPORTANCE** A capsid assembled from viral proteins protects the virus genome during transmission from one cell to another. However, when a virus enters a cell the virus genome has to be released from the capsid in order to initiate infection. This process is not well understood for nonenveloped viruses. We address this gap in our current knowledge by studying the genome release of Human Saffold virus 3. Saffold viruses cause diseases ranging from gastrointestinal disorders to meningitis. We show that before the genome is released, the Saffold virus 3 particle expands, and holes form in the previously compact capsid. These holes serve as channels for the release of the genome and small capsid proteins VP4 that in related enteroviruses facilitate subsequent transport of the virus genome into the cell cytoplasm.

## INTRODUCTION

*H*uman Saffold virus 3 (SAFV-3) belongs to the species Theilovirus in the genus Cardiovirus of the family Picornaviridae (1, 2). Cardioviruses are pathogens of vertebrates, frequently infecting rodents. The first human cardiovirus, Vilyuisk virus, was isolated in 1960 and characterized as a divergent Theiler's virus in 1992 (3). SAFV-1 was identified in a stool sample of an infant with fever (4), and SAFV-3 was isolated from the cerebrospinal fluid of a patient with aseptic meningitis (5, 6). Experimental infections of mice have shown that SAFV-3 replicates primarily in heart tissue and the central nervous system (6, 7). To date, 11 genotypes of SAFV have been identified (8–11).

Cardioviruses have nonenveloped virions with an external diameter of about 30 nm (12, 13). The icosahedral capsid contains a nonsegmented positive-sense single-stranded RNA (ssRNA) genome with a virally encoded protein VPg attached to the 5′ end and a poly(A) sequence at the 3′ end. The genome contains a single open reading frame flanked by untranslated regions (UTR) at both ends. Both the 5′ and 3′ UTRs are required for the initiation of replication. Translation of the genome is directed by an internal ribosomal entry site (IRES) within the 5′ UTR. The myristylated structural polypeptide P1 of enteroviruses is co- and posttranslationally cleaved by the viral proteases 3C and 3CD, resulting in the formation of the VP0-VP3-VP1 heterotrimeric protomer that sediments at 5S (14, 15). It was shown that 5S protomers associate into 14S pentamers and that 12 copies of 14S particles self-assemble into 80S empty capsids (16–18). The mechanism of self-assembly and the function of the empty capsids in the formation of enterovirus virions *in vivo*, however, are not well understood. In addition, active replication and translation of the poliovirus genome is required for virion formation (19). RNA-containing particles mature to virions by the cleavage of VP0 into VP2 and VP4 (20). The capsid proteins VP1, VP2, VP3, and VP4 originating from one polyprotein form a heterotetrameric protomer, an elementary building block of the capsid. The major capsid proteins VP1 to VP3 form the capsid shell with pseudo-T=3 icosahedral symmetry whereas VP4 subunits are attached to the inner surface of the capsid. VP1 subunits form pentamers around icosahedral 5-fold axes, whereas VP2 and VP3 subunits form heterohexamers positioned on icosahedral 3-fold axes. The major capsid proteins have the jellyroll β-sandwich fold common to many other virus capsid proteins.

Enteroviruses of the family Picornaviridae, which are related to cardioviruses, are extensively studied as models for genome delivery (21–26). The surface of the enterovirus capsid contains circular depressions around icosahedral 5-fold symmetry axes called “canyons.” The canyons of many enteroviruses, including major group human rhinoviruses (HRVs), are the binding sites of receptors from the immunoglobulin superfamily (27–30). However, other enteroviruses use different regions of their capsids to recognize their receptors (31, 32). Binding of receptors within the enterovirus canyon induces the release of a “pocket factor” from a hydrophobic pocket immediately below the surface of the canyon (30). Receptors that do not bind to the canyon, including LDL-R and DAF, do not induce release of the pocket factor (30–32). Furthermore, some enteroviruses, including HRV-14, lack the pocket factor even in the native state (33). In these viruses the conversion to the A particles might be induced by exposure to the low pH of the endosome (34–36). Before genome release, enterovirus virions convert into “altered” (A) particles characterized by radial expansion of the capsid and the formation of pores at the icosahedral 2-fold symmetry axes (22–25, 36–40). These changes allow the exposure of the membrane-active peptides from the capsid, which facilitates delivery of the genome into the cytoplasm. In poliovirus and CAV-A16, the genome release is accompanied by the exposure of the N-terminal region of VP1 and the release of the myristoylated VP4 (38, 41, 42).

Cardioviruses differ from enteroviruses in the details of their capsid structures and in the receptor molecules they utilize for cell entry. The structures of Mengo virus (MeV) and Theiler's murine encephalomyelitis virus (TMEV) have shown that cardiovirus virions lack canyons and pocket factors (12, 13). Some TMEV strains use glycolipids or N- and O-linked carbohydrates containing sialic acid as coreceptors (43–45). Furthermore, the murine vascular cell adhesion molecule-1 is a receptor of the D variant of an encephalomyocarditis virus (46). However, the receptor of SAFV is unknown. Despite the impact of cardioviruses on human health, we do not yet have an atomic-level structural description of SAFV virions, and the mechanism of their genome release is unknown. Here we report a crystal structure of SAFV-3 determined to a 2.5-Å resolution and a cryo-electron microscopy (cryo-EM) reconstruction of an A particle at a 10.6-Å resolution. Comparison of the two structures enabled us to propose a mechanism for the uncoating of the cardiovirus genome.

## MATERIALS AND METHODS

### SAFV-3 recovery from cDNA.

HeLa cells were transfected with the cDNA of SAFV-3 (7) using Fugene 6 according to the manufacturer's instructions. Briefly, 10 μg of SAFV-3 cDNA (7) was diluted in 168 μl of Opti-MEM medium, and 27 μl of Fugene 6 was added. The cDNA-Fugene 6 mixture was applied to a 50% confluent cell monolayer on a 50-mm-diameter plate. The cells were incubated with the transfection mixture for 2 h. Subsequently, 10 ml of minimal essential medium (MEM) was added, and the cells were grown at 37°C in a 5% CO_2_ incubator. After 48 h, a cytopathic effect (CPE) was observed, and the cells were harvested and freeze-thawed three times (at −80 and 37°C) to release the virions.

### Virus production and purification.

HeLa cells clone Ohio grown in 50 150-mm-diameter plates to 80% confluence were used for infection. The medium was aspirated from the plates, and the cells were washed with 5 ml of serum-free MEM. Cells were infected with 2 ml of the virus diluted to obtain a multiplicity of infection of 0.2 in serum-free MEM, followed by incubation for 3 h at 37°C in a 5% CO_2_ incubator with gentle shaking every 30 min. The virus titer was determined using a plaque assay on HeLa cells. Subsequently, 18 ml of MEM supplemented with 10% fetal bovine serum (FBS) and 0.2 mM l-glutamine were added to each plate, followed by incubation at 37°C. After 48 to 72 h, CPE was observed, and the cells were pelleted by centrifugation at 9,000 rpm in TA-10-250 Beckman Coulter rotor at 4°C for 15 min. After centrifugation, the cell pellet was dissolved in 10 ml of phosphate-buffered saline (PBS) and subjected to three cycles of freeze-thawing at −80 and 37°C. Subsequently, the cell debris was homogenized on ice using a Dounce tissue grinder, followed by centrifugation at 4°C at 5,000 rpm in TA-10-250 Beckman Coulter rotor for 10 min. The supernatant was mixed with the medium collected from the infected cells. The virus was precipitated from the supernatant overnight at 4°C by adding PEG 8000 and NaCl to final concentrations of 10% and 0.5 M, respectively. The solution was centrifuged at 9,000 rpm in TA-10-250 Beckman Coulter rotor at 4°C for 10 min, and the supernatant was discarded. The resulting pellet was resuspended in 10 ml of 20 mM HEPES (pH 7.5) and 150 mM NaCl at 4°C and homogenized with a Dounce tissue grinder. DNase and RNase were added to final concentrations of 10 μg/ml, and the solution was incubated at 37°C for 30 min. Subsequently, trypsin was added to a final concentration of 80 μg/ml, and the solution was incubated at 37°C for an additional 30 min, followed by centrifugation at 4,500 rpm in TA-10-250 Beckman Coulter rotor at 10°C for 10 min. The 10°C temperature was used to limit temperature changes when manipulating with the virus. The clarified supernatant was layered over 2 ml of 25% (wt/vol) sucrose cushion and centrifuged in a Beckman Ti 50.2 rotor at 48,000 rpm at 10°C for 2 h. After centrifugation, the supernatant was discarded, the pellet was resuspended in 2 ml of 20 mM HEPES (pH 7.5) and 150 mM NaCl buffer at 4°C, and the virus suspension was layered onto a 10 to 40% (wt/vol) potassium tartrate gradient prepared in the same buffer and centrifuged in an SW40 rotor at 36,000 rpm at 10°C for 90 min. The gradient layer containing the virus was collected by perforating the wall of the tube with a syringe and needle. The virus-containing fraction was transferred to 20 mM HEPES (pH 7.5) and 150 mM NaCl buffer using sequential centrifugations, and buffer additions were made using a 100-kDa cutoff Vivaspin columns (Sigma-Aldrich) at 4°C. Virus purity and concentration was verified by sodium dodecyl sulfate-gel electrophoresis. For long-term storage, the virus at 2 mg/ml was maintained at −80°C.

### Negative-stain transmission electron microscopy.

Morphology and the size of the virus particles were analyzed by transmission electron microscopy. A total of 5 μl of purified virus sample at 0.2 mg/ml was applied to a carbon-coated copper grid, allowed to adsorb for 10 min, and stained with 0.5% molybdenum acetate for 30 s. Between each transfer, excess liquid was blotted with filter paper. The grid was thoroughly dried before being mounted on a grid holder. The grids were examined, and images of viruses were captured in a Morgan Philips 201C transmission electron microscope.

### SAFV-3 crystallization and diffraction data collection.

The purified virus at a concentration of 3 mg/ml was crystallized in 2.8 M sodium acetate buffer (pH 7.0) using the hanging-drop vapor diffusion method. Rhombic crystals (∼200 μm in size) were flash cooled in liquid nitrogen and used to obtain X-ray diffraction data. The crystallization solution served as a sufficient cryoprotectant. Diffraction data were collected using a Swiss Light Source beamline X06SA and a Diamond Light Source beamline I03. Data obtained using the Swiss Light Source beamline were collected from formaldehyde-treated virus crystals.

### Structure determination.

Native SAFV-3 crystallized in space group P3_2_21. Unit cell parameters (Table 1) and packing considerations indicated that one-half of a virus particle occupied a crystallographic asymmetric unit. Plots of the 2-fold self-rotation function calculated using the program GLRF indicated that the icosahedral 2-fold axis of the virion was positioned on a crystallographic 2-fold axis (47). The locked self-rotation function had shown that the particle is rotated ϕ = 0°, φ = 90°, κ = 130.5° according to the XYK polar angle convention from the standard icosahedral orientation as defined by Rossmann and Blow (48). Reflections between resolutions of 5.0 and 4.5 Å were used for the calculations. The radius of integration was set to 140 Å. Since the particle was positioned on an icosahedral 2-fold axis, the position of the particle center had to be determined in a one-dimensional search along a line defined by *y* = 0 and *z* = 1/6. The position of the particle center was identified at the fractional coordinate *x* = 0.349 using the program Phaser from the CCP4 suite (49).

**TABLE 1:** Diffraction data and structure quality indicators^*a*^

Parameter	Native SAFV-3	DTT-treated SAFV-3
Space group	P3_2_21	P3_2_21
Unit cell dimensions		
*a, b*, *c* (Å)	300.5, 300.5, 722.1	299.9, 299.9, 723.4
α, β, γ (°)	90, 90, 120	90, 90, 120
Resolution range (Å)	70.0–2.5 (2.6–2.5)	70.0–2.5 (2.6–2.5)
No. of observations	1,540,201 (64,360)	1,606,180 (141,586)
No. of unique reflections	794,529 (43,288)	864,367 (93,415)
Observation multiplicity	1.9 (1.5)	1.9 (1.5)
Completeness (%)	61.9 (33.9)	67.5 (73.8)
*R*_merge_ (%)^*b*^	0.114 (0.505)	0.123 (0.648)
〈*I*〉/〈σ*I*〉	6.8 (1.2)	5.5 (1.0)
*R*_factor_ (%)^*c*^	22.7 (38.5)	21.5 (33.5)
No. of^*d*^:		
Protein atoms	6,012	6,012
Water molecules	343	341
Avg B factor (Å^2^)	32.8	27.6
No. of Ramachandran outliers^*e*^	2	3
RMSD		
Bond angle (°)	0.005	0.022
Bond length (Å)	1.36	1.83

a The statistics for the highest-resolution shell are shown in parentheses.

b *R*_merge_ = Σ_h_Σ_j_|l_hj_ − 〈l_h_〉|/ΣΣ|l_hj_|.

c All reflections were used in the refinement. The *R_free_* value, if it were calculated, would be very similar to R_work_ because of the 30-fold noncrystallographic symmetry present in the crystal. Therefore, the *R*_free_ would not provide an unbiased measure of model quality in this case (55). See Materials and Methods for details.

d That is, in an icosahedral asymmetric unit.

e According to the Molprobity criterion (75).

A model of the cardiovirus Theiler's murine encephalitis virus (TMEV) PDB entry 1TME converted to polyalanine was used for the molecular replacement. The model was placed into the orientation and position in the unit cell as described above and used to calculate phases for reflections up to a resolution of 10 Å using the program CNS (50). The phases were refined by 25 cycles of 30-fold real-space electron density map averaging using the program AVE (51). Phase extension was applied in order to obtain phases for higher resolution reflections. The addition of a small fraction of higher resolution data (one index at a time) was followed by three cycles of averaging. This procedure was repeated until phases were obtained for all the reflections up to a resolution of 2.5 Å. The model was built using the programs O and Coot (52, 53), starting from the TMEV coordinates where the respective residues were mutated to those of SAFV-3. The model was refined by manual rebuilding, alternating with coordinate refinement using the program CNS (simulated annealing, gradient minimization, and individual B-factor refinement) (50). Other calculations were carried out using CCP4 (54). All the measured reflections were used in the refinement. If calculated, *R*_free_ would be very similar to the *R* value due to the 30-fold noncrystallographic symmetry (NCS) present in the diffraction data (55). Reflections related by the NCS present in the crystal and diffraction data are correlated with each other. This correlation was utilized in the phase extension; however, because of the correlation it was not possible to select reflections in the *R*_free_ test set that were not correlated with the working set reflections used in the refinement. It was demonstrated previously that in the presence of 30-fold NCS, even the use of thin shells or symmetry-related groups of reflections for the test set is not sufficient (56). Crystals of dithiothreitol (DTT)-treated SAFV-3 were of the same space group as those of the native virus but had slightly different unit cell parameters. The structure was determined in the same manner as the native one; however, the native model of SAFV-3 was used for the molecular replacement.

### Induction of SAFV-3 genome release by heating.

The stability of SAFV-3 was determined as the temperature at which 50% of its RNA genome was accessible to fluorescent RNA-binding dye Sybr green II. Virions at a concentration of 0.02 mg/ml in 0.25 M HEPES (pH 7.5)–0.25 M NaCl buffer were incubated with Sybr green II (diluted ×3,000 times from the stock solution according to the manufacturer's instructions), and the mixture was heated from 25 to 95°C in 1°C increments with a 2-min incubation time at each temperature in the real-time PCR instrument (Roche LightCycler 480). The fluorescence signal increases as the dye interacts with RNA that is released from the thermally destabilized particles, or the dye might be able to enter the particles. The thermal stability of the virus was estimated at the temperature corresponding to an increase in the fluorescence to 50% of the maximal value obtained when all virions were thermally denatured. The measurements were carried out in triplicate.

### Determination of the effect of DTT on SAFV-3 infectivity.

The effect of DTT on the infectivity of SAFV-3 was quantified by flow cytometry using propidium iodide dye to stain dead and apoptotic cells. DTT at a final concentration of 10 mM was added to SAFV-3 and EV71 in MEM supplemented with 10% serum and 0.2 mM l-glutamine, followed by incubation for 15 min at room temperature. The DTT was removed from the virus by repeated medium exchange in Vivaspin concentrators by concentrating the virus to 1/10 of the initial volume and diluting the samples three times. The control virus stocks were treated in the same way except for the absence of DTT. Prior to virus infection, HeLa cells grown to 80% confluence grown in a six-well dish were washed with 2 ml of serum-free MEM. The cells were incubated at 37°C for 2 h with 200 μl of the DTT-treated and control untreated viruses. The medium was removed and 2 ml of fresh MEM supplemented with 10% FBS and 0.2 mM l-glutamine were added to the cells, followed by incubation at 37°C. After 24 h, the cells from one plate were washed with PBS, trypsinized into 0.1 ml of final volume of PBS, and stained with 1 μl of 1 mg/ml propidium iodide dissolved in water for 5 min. The number of dead cells was determined using a flow cytometer (BD FACSVerse). All experiments were performed in triplicates.

### Cryo-EM data collection and structure determination.

SAFV-3 solution with a concentration of 2 mg/ml in 20 mM HEPES (pH 7.5) and 150 mM NaCl buffer was heated for 2 min at 42°C to produce A particles, applied to a holey carbon film, blotted, and immediately vitrified by plunging into liquid ethane using Vitrobot Mark 4. The use of a 2-s blotting time with force −2 resulted in optimal ice thickness.

Electron micrographs were recorded with a BM Eagle CCD detector in a FEI TF20 FEG microscope using defocus ranging from −2.33 to −3.95 μm. The micrographs had a pixel size of 2.22 Å. In total, 17,483 particles were boxed out from the micrographs using the program e2boxer.py, and the defocus of each micrograph was determined using the program e2ctf.py from the EMAN2 suite (57). Particle images were phase corrected for the effect of the contrast transfer function using the program fitctf2.py (58). Particles were separated into two halves (odd and even image numbers) and treated completely independently in according to the gold standard approach (59). First, nine sets of 400 particles were randomly selected from each half of the data and assigned random orientation parameters for initial reconstruction using the program j3dr (59). Icosahedral symmetry was imposed during the reconstruction. The orientation parameters were then refined using template matching within the icosahedral asymmetric unit in the program jalign (59). Independent initial models were selected for the even and odd data sets, and the phases of these initial models were randomized below a resolution of 40 Å. Multiple independent alignment reconstruction steps were iterated to select bad particles based on their inconsistency in the assignment of orientations. Finally, the orientation parameters for 7,014 selected particles in each half of the data were further refined to produce final reconstructions for the even and odd data sets. The resolution of the resulting map was estimated as the resolution at which the correlation between the two sets of structure factors derived from the two reconstructions fell below 0.143.

### Fitting native SAFV-3 model into cryo-EM reconstruction of empty particle.

The electron density map of VP1, VP2, and VP3 derived from the native structure of SAFV-3 was manually positioned into the cryo-EM map of the A particle using the program VMD (60), and its position was refined using the program Chimera (61). A “difference map” was calculated by setting to zero the density values of grid points within 5 Å of any VP1, VP2, or VP3 atoms for all but one of the subunits at a time. The crystal structures of VP1, VP2, and VP3 were consequently fitted into the corresponding difference maps using the multifragment refinement program Collage (62). New difference maps were calculated based on the updated positions of VP1, VP2, and VP3, and the fitting was repeated iteratively. A convergence was reached in four repeats of the fitting. The resulting positions of VP1, VP2, and VP3 determined by multifragment rigid-body fitting in the program Collage achieved a 5% increase in the correlation coefficient, with 98% of the atoms contained within the map.

### Accession number(s).

Protein Data Bank (PDB) models of native and DTT-treated SAFV-3, together with structure factor amplitudes and phases derived by phase extension, have been deposited in the PDB under codes 5CFC and 5CFD, respectively. A cryo-EM reconstruction map of the SAFV-3 A particle was deposited with EMDB under number EMD-3097 (http://emsearch.rutgers.edu/atlas/3097_summary.html), and the fitted coordinates were deposited in the PDB under code 5A8F.

## RESULTS AND DISCUSSION

### Structure of the SAFV-3 virion and capsid proteins.

The crystal structure of the SAFV-3 virion was determined to a resolution of 2.5 Å. Each of the major capsid proteins, VP1, VP2, and VP3, has a β-sandwich fold that consists of eight β-strands, conventionally named B-I, forming the two antiparallel β-sheets containing strands BIDG and CHEF (12, 13, 33, 63). The N termini of the major capsid proteins are located on the inside the capsid, while the C termini are exposed on the particle surface. The electron density map enabled residues 1 to 260 of VP1, 11 to 268 of VP2, and 1 to 232 of VP3 to be built. The VP4 protein of SAFV-3 contains 72 residues; however, only residues 15 to 38 could be built. Crystallographic diffraction data and structure quality indicators are listed in Table 1.

**FIG 1:**
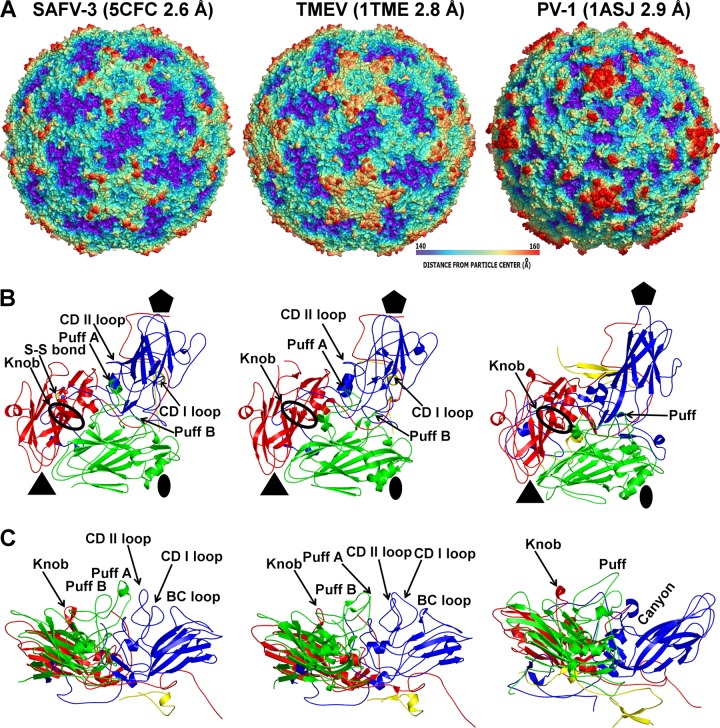
Comparison of SAFV-3, TMEV, and poliovirus 1 capsid protein and virion structures. (A) Surface rendering of virions rainbow-colored based on distance from particle center. (B) Diagrams of icosahedral asymmetric units. VP1 is shown in blue, VP2 is shown in green, VP3 is shown in red, and VP4 is shown in yellow. The positions of the prominent surface features “puff” in VP2 and “knob” in VP3 are indicated. Cys87 and cys89 forming a disulfide bond in SAFV-3 VP3 are shown in stick representation. (C) Side views of icosahedral asymmetric units demonstrating the absence of a canyon in SAFV-3.

The surface of the SAFV-3 virion is characterized by the presence of star-shaped plateaus around icosahedral 5-fold axes and depressions located around icosahedral 2-fold axes (Fig. 1A). The highest protrusions on the virus surface are formed by the BC and CD loops of VP1 positioned at the border of the arms of the star-shaped plateau and the edges of the 2-fold depressions (Fig. 1). In place of a continuous canyon, as seen in enteroviruses (33, 63), there are five pits around each 5-fold axis in SAFV-3 (Fig. 1A). The CD loop of VP1 fills the volume that corresponds to the canyon in enteroviruses (Fig. 1B and C). The EF loop of VP2 (residues 132 to 195), which is by convention named “puff,” is subdivided into two loops, puff A and B, in the SAFV-3 virion (Fig. 1B and C). Puff B contains a short α helix that has not been observed in other cardioviruses (Fig. 1B and C) (12, 13). Residues from puff B interact with the CD loop of VP1, and the short α helix is located on the surface of the virion (Fig. 1B and C). Structures resembling puff B are not present in enteroviruses (Fig. 1B and C) (33, 63). The most prominent feature on the capsid surface formed by VP3 is a finger-like protrusion conventionally named a “knob,” which in SAFV-3 contains two β-strands (Fig. 1C). Similar short β-strands have been previously observed in knobs of TMEV and MeV (Fig. 1B and C) (12, 13). In enteroviruses the knob is usually five residues shorter and lacks the β-strands (Fig. 1B and C) (33, 63). However, the knob and puff regions were implicated in the receptor binding of echovirus 7 to its cellular receptor DAF (31). RMSD values comparing the distances between equivalent Cα atoms of icosahedral asymmetric units of SAFV-3 with selected picornaviruses are listed in Table 2. Overall, the architecture of SAFV-3 is most similar to that of TMEV (13).

**TABLE 2:** Sequence and structural similarities of capsid proteins of selected picornaviruses

Virus	RMSD (Å) and % identity^*a*^										
	SAFV3	TMEV	EMCV	PV1	HRV14	HAV	HPeV1	EV71	EV7	CVB3	CVA16
SAFV3		0.8	1.1	1.5	1.5	2.0	2.1	1.5	2.7	1.6	1.6
TMEV	70		1.0	1.5	1.5	2.0	2.1	1.7	1.5	1.5	1.6
EMCV	60	64		1.8	1.7	2.6	2.6	1.9	1.7	1.7	1.8
PV1	29	29	28		1.0	2.1	2.1	1.1	0.9	1.0	1.0
HRV14	25	26	28	49		2.1	2.1	1.1	1.1	1.0	1.2
HAV	20	21	18	18	19		2.1	2.1	2.1	2.0	2.1
HPeV1	19	20	19	17	17	18		2.2	2.1	3.0	2.2
EV71	29	30	29	44	44	17	15		1.0	1.0	0.5
EV7	28	29	27	55	47	19	17	46		0.7	1.0
CVB3	28	29	28	54	50	20	10	48	72		1.0
CVA16	30	30	29	47	44	17	15	79	48	49	

a The top right portion of the table shows the root mean square deviations (RMSD) of superimposed Cα atoms of the respective three-dimensional structures. Capsid protein protomers corresponding to icosahedral asymmetric units consisting of subunits VP1 to VP4 were used in the comparisons. The program Coot was used for superposition of the molecules (53). In the bottom left portion of the table are the percent identities between the respective virus coat protein sequences. Gaps were ignored in the calculations.

**FIG 2:**
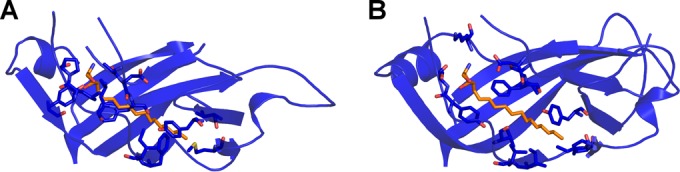
VP1 of SAFV-3 does not contain a hydrophobic pocket. (A and B) Diagrams of VP1 of SAFV-3 (A) and poliovirus 1 (B). The pocket factor in poliovirus 1 is shown as a stick model in orange. Residues that interact with the pocket factor are also shown as sticks. In panel A, the poliovirus pocket factor was superimposed onto the structure of SAFV-3. However, the pocket is not formed in SAFV-3 VP1, and the side chains of several residues clash with the pocket factor.

The space between the two β-sheets of VP1, which in some enteroviruses contains a hydrophobic cavity with a pocket factor, is filled by the side chains of residues forming the core of the protein in SAFV-3 (Fig. 2). Thus, it is unlikely that pocket binding inhibitors that can prevent genome release from enteroviruses (64, 65) could be used to inhibit SAFV infections.

### The disulfide bond in the surface loop of VP3 and its role in cardiovirus infectivity.

**FIG 3:**
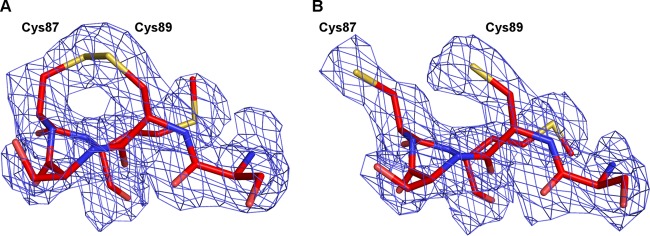
Disruption of VP3 Cys87-Cys89 disulfide bond results in reorientation of cysteine side chains; however, it has limited impact on VP3 structure. (A and B) Detail diagram of the CD loop in VP3 of SAFV-3 in native virus (A) and DTT-treated virus (B). An electron density map contoured at 2σ is shown as a blue mesh.

SAFV-3 contains a disulfide bond connecting cys87 and cys89 in the CD loop of VP3 (Fig. 3A). Disulfide bonds connecting side chains of cysteines that are separated by a single residue are uncommon, because such links introduce sharp bends in the polyprotein main chain (12, 13). The CD loop of VP3 is located on the surface of the virus close to the knob region (Fig. 1B). The two cysteine residues are conserved among SAFVs, except for SAFV-5 and SAFV-6, and also in homologous positions in the MeV and TMEV capsids. The disulfide bond was observed in the MeV (12) but not in TMEV (13). It was suggested by Grant et al. that the disulfide bond in TMEV was disrupted because of covalent modifications of the cysteine side chains that occurred during virus purification or crystallization (13).

**FIG 4:**
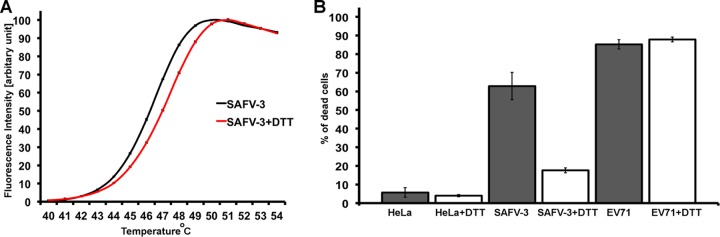
Impact of disruption of the Cys87-Cys89 disulfide bond on stability and infectivity of SAFV-3. (A) A Sybr green fluorescence assay was performed to measure the stability of SAFV-3 particles. SAFV-3 virions were mixed with Sybr green dye II and heated to the indicated temperatures (*x* axis). The fluorescence signal increases as the dye binds to RNA that is released from thermally destabilized particles. The black line represents native SAFV-3 virions, and the red line represents SAFV-3 treated with 10 mM DTT. Error bars indicate the standard deviations of the measurements. See Materials and Methods for additional details. (B) Effect of DTT treatment on infectivity of SAFV-3 and EV71. Fractions of dead cells were determined based on propidium iodide staining.

In order to investigate the importance of the disulfide bond for maintaining the native structure and infectivity, the SAFV-3 virions were crystallized in reducing conditions containing 10 mM DTT. Crystals of DTT-treated SAFV-3 virions diffracted to a resolution of 2.5 Å. A comparison to the native SAFV-3 virion showed that disruption of the Cys87-Cys89 disulfide bond had minimal effect on the structure of the VP3 CD-loop (Fig. 3) and no effect on the other parts of the virion. The root mean square deviation (RMSD) of equivalent atoms in the two SAFV structures is 0.062 Å. The average crystallographic temperature factors of atoms of residues 86 to 89 of the CD loop of VP3 were 38.1 Å^2^ in the native structure and 40.7 Å^2^ in the structure with the disrupted disulfide bond. Similar values of temperature factors indicate that the disruption of the disulfide bond did not increase the flexibility of the loop. However, disruption of the disulfide bond by DTT increased the stability of the capsid by 2°C, measured as the temperature at which the virions release their RNA genomes (Fig. 4A). A similar increase in temperature stability has been previously reported for enterovirus virions upon binding of WIN compounds (66). This DTT-induced increase in virion stability is not associated with structural changes at 22°C, the temperature at which the SAFV-3 crystals were grown. However, it is possible that at temperatures in the range of 48 to 52°C, the disruption of the disulfide bond results in an alteration of capsid dynamics, so-called “capsid breathing” (67), that might allow the virions to retain their genomes at higher temperatures. Furthermore, the reduction of the bond resulted in a 70% decrease in the infectivity of SAFV-3 (Fig. 4B). In contrast, a similar decrease in infectivity was not observed for EV71, which does not contain disulfide bonds in the capsid (Fig. 4B). Conservation of the cysteine residues in loops exposed on the cardiovirus virion surface indicates a possible role of the disulfide bond in virus infection. A putative explanation of the reduced infectivity of SAFV-3 virions with the disrupted disulfide bond might be that residues 86 to 89 of the VP3 CD loop play a role in receptor binding. The disulfide bond could be important for retaining the optimal conformation of the loop for binding to the receptor. Alternatively, the increased stability of the virions caused by the disruption of the disulfide bond might decrease the efficiency of genome release from virions (21, 26, 38, 68); however, we did not observe differences in genome release between native and DTT-treated virus (data not shown). Previous work on MeV demonstrated that addition of 0.1% β-mercaptoethanol increased its infectivity by reducing abortive infection of mouse fibroblasts (69). Furthermore, heat stability of many picornaviruses can be increased by interaction with sulfhydryl reducing groups (70). The effects of reducing agents indicate that compounds specifically disrupting or binding to the disulfide Cys87-Cys89 bond might reduce SAFV infectivity.

### Cardiovirus genome release results in formation of unstable empty capsids that disassemble into pentamers.

In order to initiate infection, virus genomes need to be released from virions and transferred across the biological membrane into the cell cytoplasm. For most eukaryotic nonenveloped viruses, this process is poorly understood. However, enteroviruses from the family Picornaviridae have been extensively studied as model organisms for genome delivery into the cytoplasm (21–25, 37, 71). The genome release of enteroviruses is preceded by structural changes to the capsid, leading to the formation of an expanded A particle that is induced by receptor binding and/or by the low pH of late endosomes (21, 26, 38, 68). Similar structural changes to particles can be induced *in vitro* by heating enterovirus virions to nonphysiological temperatures of about 56°C (37, 71). After the genome release, enterovirus capsids remain stable, at least *in vitro*, forming empty “B” particles (72).

**FIG 5:**
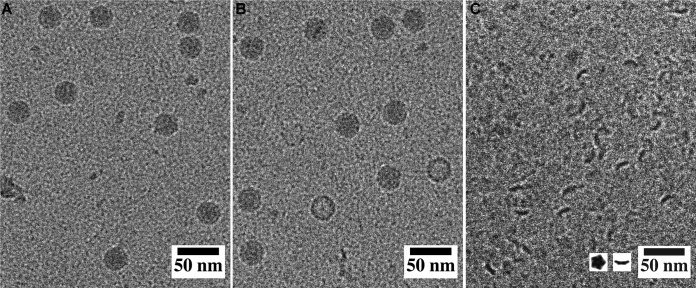
Release of SAFV-3 genome results in the formation of empty particles that disintegrate into pentamers. (A) Cryo-EM images of native virions. (B) SAFV-3 particles heated to 42°C for 2 min. The sample contained A particles and a few empty capsids. (C) Pentamers of capsid protein protomers prepared by incubating SAFV-3 virions at 42°C for 2 min and at 23°C for 5 min. The inset in panel C shows top and side view projections of pentamers of SAFV-3 protomers calculated from the structure of capsid proteins.

The formation of SAFV-3 A particles can be induced by heating the virions to 42°C for 2 min. In addition to A particles, the sample also contained about 1% of empty capsids (Fig. 5B). However, if SAFV-3 virions were left at room temperature for more than 5 min after heating, the sample contained almost exclusively pentamers of capsid protein protomers (Fig. 5C). Native protein electrophoresis combined with mass spectrometry analysis verified that Safv-3 virions disassembled into pentamers after heating to 42°C, followed by cooling to 25°C (data not shown). This corroborates the previous suggestion that cardiovirus virions disassemble upon or shortly after genome release (34, 73). However, RNA uncoating of the related MeV is triggered at 37°C upon cell attachment, possibly leaving behind an unstable empty capsid, which subsequently disintegrates into 14S pentamers (69). We show that *in vitro* after being heated to 42°C, the SAFV-3 virions convert to A particles and release their genomes, and the resulting empty capsids rapidly dissociate into pentamers.

### The SAFV-3 A particle contains pores that enable the externalization of VP1 N termini and of VP4 subunits.

**FIG 6:**
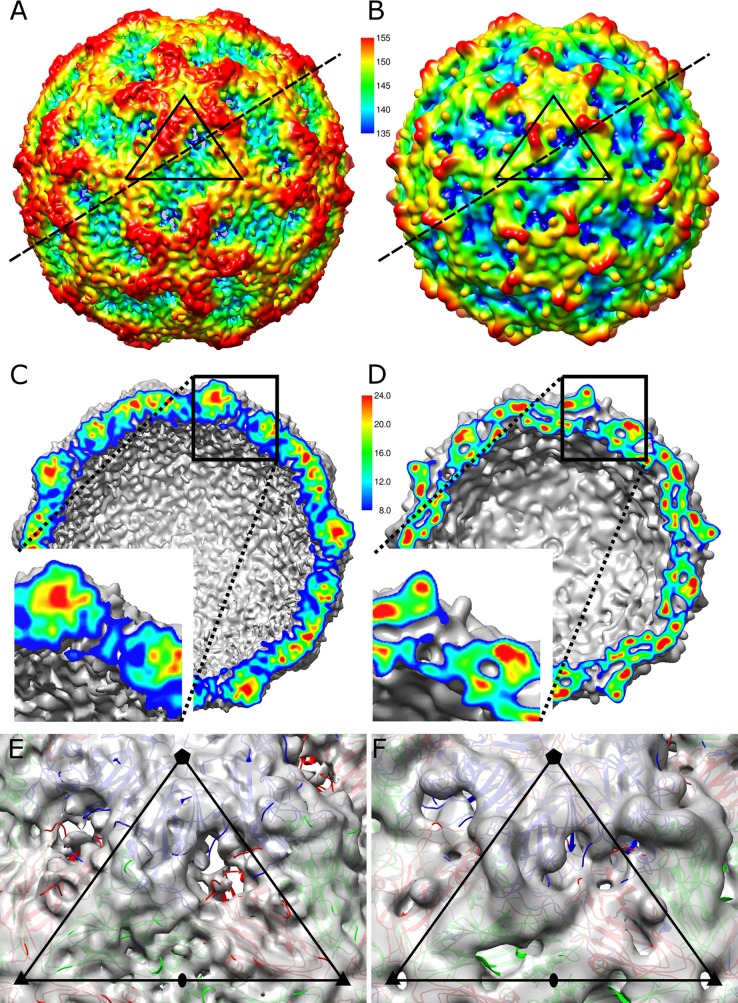
Native SAFV-3 virion has compact capsid, whereas A particle contains pores. (A and B) Surface renderings of SAFV-3 A particle (A) and native virion (B) at a 10.6-Å resolution. The surfaces displayed at 0.5σ are rainbow-colored based on the distance from the particle center, as indicated by the scale bar. Distances are indicated in angstroms. The borders of a selected icosahedral asymmetric unit are indicated with a black triangle. Dashed lines indicate the planes of cross sections through the particles, as displayed in panels C and D. (C and D) Cross-section views of SAFV-3 A particle (C) and native virion (D). The insets show enlarged regions of the cross sections corresponding to the opening in the capsid of the A particle. The cross sections are colored according to electron density, as indicated by the scale bar in arbitrary units. (E and F) Details of icosahedral asymmetric units of A particle (E) and native virus (F). Electron densities displayed at 1σ are shown as semitransparent surfaces. VP1 subunits are shown in blue, VP2 subunits are shown in green, and VP3 subunits are shown in red. The borders of the icosahedral asymmetric unit are highlighted as black triangles, and the positions of 5-fold, 3-fold, and 2-fold icosahedral symmetry axes are indicated by pentagons, triangles, and ovals, respectively.

The enterovirus A particle represents a virion state that not only is primed for genome release but also facilitates the transport of the genome across a biological membrane. This function is ensured by the release of VP4 subunits that make cellular membranes permeable for viral RNA and by externalization of the N-terminal arms of VP1, which contain hydrophobic residues that, in some viruses, anchor virions to membranes (38). Here, we present a 10.6-Å resolution cryo-EM reconstruction of the SAFV-3 A particle and show that it is expanded 4% in diameter compared to the native virion (Fig. 6A and B). Capsids of SAFV-3 A particles have pores located approximately in the middle between the 5-fold and 3-fold icosahedral symmetry axes (Fig. 6A and C) at the interface between two VP1 subunits related by a 5-fold symmetry axis and a VP3 subunit (Fig. 6E). The pores are circular in shape with a diameter of ∼15 Å. Models of the capsid protein subunits determined in the native SAFV-3 structure were fitted as rigid bodies into the native cryo-EM density map of the A particle. The formation of the pore is caused by shifts of the VP1 subunits toward the icosahedral 5-fold axis and of VP3 toward the icosahedral 3-fold axis relative to their positions in the native virus (Fig. 6E and F). The changes in subunit positions are possible due to the radial expansion of the capsid. However, parts of the VP1 and VP3 subunits located close to the pore did not entirely fit into the cryo-EM electron density map (Fig. 6C and E). Low values of density at the border of the pore indicate that the EF loop and C terminus of VP1 and CD, GH loops and C terminus of VP3, which are the located in the vicinity of the pore, are flexible.

**FIG 7:**
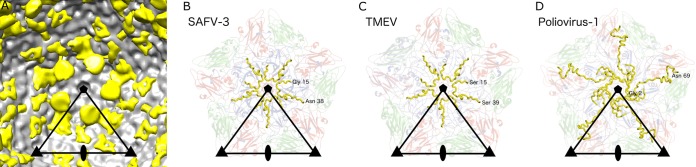
SAFV-3 A particles lack capsid protein VP4. (A) The cryo-EM density of SAFV-3 A particle displayed at 1σ is shown in gray, and a difference map calculated by subtracting the electron density of A particle from that of the native virus is shown in yellow. The yellow difference density displayed at 1σ corresponds to VP4 in the native particle. (B to D) Positions of VP4 subunits in SAFV-3 (B), TMEV (C), and poliovirus 1 (D) virions. The pentamers of capsid protein protomers are shown. VP4 subunits are shown in yellow, VP1 subunits are shown in blue, VP2 subunits are shown in green, and VP3 subunits are shown in red. The labels indicate the type and number of the first and last structured residues of VP4 in the respective models.

A difference map calculated by subtracting the electron density map of the SAFV-3 A particle from that of the native virion indicates that VP4 is missing from the A particle (Fig. 7). The last ordered residues of the SAFV-3 VP4 are located at contacts between two 5-fold related VP1 subunits and VP3, immediately below the opening of the pore in the A particle (Fig. 7B). In contrast to the HRV-14 and poliovirus virions, the ordered part of SAFV-3 VP4 begins close to a 5-fold axis and ends at a 3-fold axis of the same protomer (Fig. 7D). The flexible part of VP4, not visible in the native SAFV-3 virion, is thus optimally positioned for release from the A particle.

### Structural differences between cardiovirus and enterovirus A particles.

**FIG 8:**
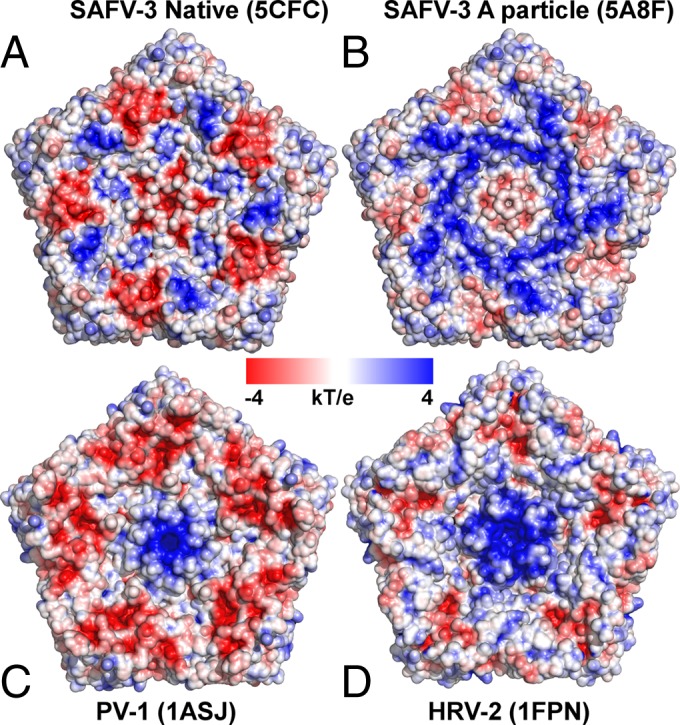
Charge distributions inside picornavirus capsids. (A and B) Charge distributions inside native SAFV-3 virion (A) and A particle (B). (C and D) Charge distributions inside poliovirus 1 (C) and HRV-2 (D). Pentamers of capsid protein protomers are shown.

The structure of the A particle of SAFV-3 is different from that of enteroviruses. “A” particles of EV71, poliovirus, HRV-2, CAV-7, and CAV-16 contain two types of pores located at (i) icosahedral 2-fold axes and (ii) between icosahedral 2-fold axes and 5-fold axes (22–25, 36–40). In the CAV-16 A particle, the pores at the 2-fold axes were proposed to be locations for the externalization of VP1 N termini that are then translocated to the channels between the 2-fold and 5-fold axes (39). In addition, the pores at 2-fold axes were proposed to serve as channels for the release of VP4 subunits and the RNA genome. However, the channels in enterovirus A particles had to expand in order to allow the passage of ssRNA (39, 74). The borders of the channels located at 2-fold axes in enterovirus particles are formed by helix α3 from the CD loop of VP2 and EF loop of VP3 (39, 74). The overall distribution of charges inside the SAFV-3 and enterovirus capsids does not exhibit differences that could explain the different stabilities of their empty capsids (Fig. 8).

SAFV-3 A particles do not contain pores at icosahedral 2-fold symmetry axes (Fig. 6A and E). However, the pores located between the 3-fold and 5-fold axes are larger than either of the pores observed in enterovirus A particles. There is a higher density in the center of the pore in the SAFV-3 A particle that is not connected to the surrounding electron density of the capsid (Fig. 6C). The unconnected density might belong to VP1 N termini passing through the pore from inside the virion to outside the virion. Presumably, VP1 N termini are flexible or adapt various conformations both inside and outside the capsid and therefore are not visible in the cryo-EM reconstruction that corresponds to the average of many particles. However, the movements of the VP1 N-terminal arms are restricted to the pore center, resulting in the observed density (Fig. 6C and E).

### Structure of the genome in Safv-3 virions and A particles.

**FIG 9:**
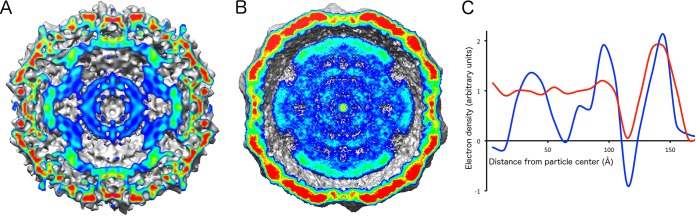
(A and B) Electron density of RNA inside SAFV-3 native virion (A) and A particle (B). The central sections of the particles are shown. The cross sections are colored according to electron density values. (C) Spherically averaged electron density distribution of native SAFV-3 virion (blue) and A particle (red).

The conversion of SAFV-3 virions to A particles not only affects the structure of the capsid but also the structure of the RNA genome. In the native virus, the RNA is distributed in two spherical shells of density located 35 and 95 Å from the particle center (Fig. 9A). Parts of the genome located around 5-fold and 2-fold icosahedral axes of the particle appear to be in contact with the capsid. It was previously speculated that the N-terminal parts of major capsid proteins VP1 to VP3 that are not resolved in the structure are in direct contact with the genome. In contrast, in the A particle the RNA density is distributed uniformly at the central part of the virion within a sphere with a radius of 105 Å (Fig. 9B). The RNA does not form any contacts with the capsid of the A particle, unlike in the previously studied A particles of enteroviruses (25, 39). The disruption of the capsid RNA contacts might be due to the changes in the distribution of charges on the inner face of the capsid associated with the release of VP4 subunits and externalization of VP1 N termini from the particle. However, the overall charge of the inner face of the A particle is more positive than that of the native virion (Fig. 8). The disruption of the interactions of the capsid with the genome upon conversion of the virion to the A particle might facilitate subsequent release of the RNA from the capsid.

### Genome release from the SAFV-3 A particle.

Expansion of the SAFV-3 capsid upon formation of the A particle results in an increase in the interior volume of the virion from 5.3 × 10^6^ Å^3^ to 6.8 × 10^6^ Å^3^. This expansion might be required to increase the mobility of the genome within the particle, which might be necessary to enable the 3′ end of the RNA to find one of the pores within the A particle before it can be released from the capsid (71).

Observations of empty SAFV-3 particles immediately after heating to 42°C (Fig. 5B and C) indicate that the genome is released from A particles that then disassemble into pentamers. The pores in the capsid of the A particles are sufficiently large to allow passage of single-stranded RNA. However, the genome contains RNA sequences that form functional double-stranded regions, which are components of the IRES and stem-loop structures required for picornavirus translation and replication. It is possible that these RNA secondary structure elements are retained when the genome is packaged in the virion. If this was the case, the genome would either have to unwind or the pore serving for genome release would have to be expanded in order to allow passage of the RNA. The flexible nature of the loops surrounding the pore (Fig. 6C) indicates that pore enlargement might be possible. Thus, compounds stabilizing or blocking the pores in the A particle might serve as tools for further study of cardiovirus genome release and might be developed into SAFV inhibitors.
